# Supplementary material for: A Norrin/Wnt surrogate antibody stimulates endothelial cell barrier function and rescues retinopathy
Source: EMBO Mol Med. 2021 Jun 9;13(7):e13977. doi: 10.15252/emmm.202113977 (PMC8261507; doi:10.15252/emmm.202113977)
Supplement: Supplementary file 1 — Appendix [file EMMM-13-e13977-s005.pdf]

Table Appendix S1: Exact p values

ANOVA + Bonferroni's = Ordinary one-way ANOVA with Bonferroni's multiple comparisons test

T-test = Student's unpaired t-test

	Phospho-DVL3 Western blots		
figure 1E	ANOVA + Bonferroni's	Summary	Adjusted P Value
	Non-treated vs. F4L5.13 15min	ns	0.4366
	Non-treated vs. F4L5.13 30min	ns	0.0643
	Non-treated vs. F4L5.13 60min	*	0.0364
	Non-treated vs. NDP 15min	*	0.0354
	Non-treated vs. NDP 30min	****	<0.0001
	Non-treated vs. NDP 60min	****	<0.0001
	ßcatenin Western blots		
	ANOVA + Bonferroni's	Summary	Adjusted P Value
	Non-treated vs. F4L5.13 15min	ns	>0.9999
	Non-treated vs. F4L5.13 30min	*	0.0123
	Non-treated vs. F4L5.13 60min	ns	0.3652
	Non-treated vs. NDP 15min	ns	>0.9999
	Non-treated vs. NDP 30min	ns	0.4215
	Non-treated vs. NDP 60min	ns	>0.9999

	Figure 3C left		
	ZO-1 IF quantification		
Figure 3C	ANOVA + Bonferroni's	Summary	Adjusted P Value
	Non-treated vs. VEGF	****	<0.0001
	Non-treated vs. VEGF + F4L5.13	ns	>0.9999
	Non-treated vs. VEGF + NDP	ns	>0.9999
	VEGF vs. VEGF + F4L5.13	****	<0.0001
	VEGF vs. VEGF + NDP	****	<0.0001
	VEGF + F4L5.13 vs. VEGF + NDP	ns	>0.9999
	CLDN5 IF quantification		
	ANOVA + Bonferroni's	Summary	Adjusted P Value
	Non-treated vs. VEGF	****	<0.0001
	Non-treated vs. VEGF + F4L5.13	ns	0.8923
	Non-treated vs. VEGF + NDP	ns	>0.9999
	VEGF vs. VEGF + F4L5.13	***	0.0003
	VEGF vs. VEGF + NDP	****	<0.0001
	VEGF + F4L5.13 vs. VEGF + NDP	ns	>0.9999

	Figure 3C left		
	ZO-1 IF quantification		
Figure 3C	ANOVA + Bonferroni's	Summary	Adjusted P Value
	Non-treated vs. VEGF	**	0.004
	Non-treated vs. VEGF + F4L5.13	*	0.0322
	Non-treated vs. VEGF + NDP	ns	>0.9999
	VEGF vs. VEGF + F4L5.13	****	<0.0001
	VEGF vs. VEGF + NDP	*	0.0215
	VEGF + F4L5.13 vs. VEGF + NDP	**	0.0063
	CLDN5 IF quantification		
	ANOVA + Bonferroni's	Summary	Adjusted P Value
	Non-treated vs. VEGF	***	0.0002
	Non-treated vs. VEGF + F4L5.13	ns	>0.9999
	Non-treated vs. VEGF + NDP	ns	>0.9999
	VEGF vs. VEGF + F4L5.13	****	<0.0001
	VEGF vs. VEGF + NDP	**	0.0013
	VEGF + F4L5.13 vs. VEGF + NDP	ns	0.4907

	Permeability assay		
Figure 3D	ANOVA + Bonferroni's	Summary	Adjusted P Value
	Non-treated vs. VEGF	****	<0.0001
	Non-treated vs. F4L5.13	ns	>0.9999
	Non-treated vs. VEGF+F4L5.13	ns	>0.9999
	Non-treated vs. Pre-VEGF+F4L5.13	ns	0.4248
	VEGF vs. VEGF+F4L5.13	****	<0.0001
	VEGF vs. Pre-VEGF+F4L5.13	****	<0.0001

	ANOVA + Bonferroni's	Summary	Adjusted P Value
figure 4D	WT vs. KO Isotype control	****	<0.0001
	WT vs. KO F4L5.13	ns	>0.9999
	KO Isotype control vs. KO F4L5.13	****	<0.0001

	ANOVA + Bonferroni's	Summary	Adjusted P Value
Figure 5B	WT vs. KO Isotype control	****	<0.0001
	WT vs. KO F4L5.13	***	0.0008
	KO Isotype control vs. KO F4L5.13	**	0.0044

	T-Test	Summary	pvalue
Figure 5C	KO Isotype control vs. KO F4L5.13	ns	0.3649

	Figure EV4		
	**ANOVA + Bonferroni's**	**Summary**	**Adjusted P Value**
Bmp2	Non-treated vs. F4L5.13 8h	ns	0.1847
	Non-treated vs. F4L5.13 24h	****	<0.0001
	Non-treated vs. NDP 8h	ns	0.6017
	Non-treated vs. NDP 24h	*	0.0172
Ang-2	Non-treated vs. F4L5.13 8h	ns	>0.9999
	Non-treated vs. F4L5.13 24h	**	0.0045
	Non-treated vs. NDP 8h	ns	>0.9999
	Non-treated vs. NDP 24h	ns	0.6061
Jag2	Non-treated vs. F4L5.13 8h	ns	0.1114
	Non-treated vs. F4L5.13 24h	**	0.0014
	Non-treated vs. NDP 8h	ns	>0.9999
	Non-treated vs. NDP 24h	*	0.017
Axin2	Non-treated vs. F4L5.13 8h	*	0.0294
	Non-treated vs. F4L5.13 24h	**	0.0014
	Non-treated vs. NDP 8h	ns	>0.9999
	Non-treated vs. NDP 24h	ns	0.3689
Mal	Non-treated vs. F4L5.13 8h	ns	>0.9999
	Non-treated vs. F4L5.13 24h	**	0.0071
	Non-treated vs. NDP 8h	ns	>0.9999
	Non-treated vs. NDP 24h	ns	>0.9999
Scin	Non-treated vs. F4L5.13 8h	ns	0.246
	Non-treated vs. F4L5.13 24h	**	0.0017
	Non-treated vs. NDP 8h	ns	0.8119
	Non-treated vs. NDP 24h	ns	0.1026
Nfatc2	Non-treated vs. F4L5.13 8h	****	<0.0001
	Non-treated vs. F4L5.13 24h	****	<0.0001
	Non-treated vs. NDP 8h	**	0.0048
	Non-treated vs. NDP 24h	***	0.0002
Rerg	Non-treated vs. F4L5.13 8h	ns	0.0677
	Non-treated vs. F4L5.13 24h	***	0.0002
	Non-treated vs. NDP 8h	ns	0.9532
	Non-treated vs. NDP 24h	ns	0.3969
Zo-1	Non-treated vs. F4L5.13 8h	ns	>0.9999
	Non-treated vs. F4L5.13 24h	ns	>0.9999
	Non-treated vs. NDP 8h	ns	>0.9999
	Non-treated vs. NDP 24h	ns	>0.9999

Cldn5	Non-treated vs. F4L5.13 8h	ns	0.9422
	Non-treated vs. F4L5.13 24h	ns	>0.9999
	Non-treated vs. NDP 8h	ns	0.3868
	Non-treated vs. NDP 24h	ns	>0.9999
Cldn3	Non-treated vs. F4L5.13 8h	ns	>0.9999
	Non-treated vs. F4L5.13 24h	ns	>0.9999
	Non-treated vs. NDP 8h	ns	>0.9999
	Non-treated vs. NDP 24h	ns	>0.9999
Cdh5	Non-treated vs. F4L5.13 8h	ns	>0.9999
	Non-treated vs. F4L5.13 24h	ns	>0.9999
	Non-treated vs. NDP 8h	ns	0.0671
	Non-treated vs. NDP 24h	ns	>0.9999
