# Supplementary material for: A Norrin/Wnt surrogate antibody stimulates endothelial cell barrier function and rescues retinopathy
Source: EMBO Mol Med. 2021 Jun 9;13(7):e13977. doi: 10.15252/emmm.202113977 (PMC8261507; doi:10.15252/emmm.202113977)
Supplement: Supplementary file 2 — Expanded View Figures PDF [file EMMM-13-e13977-s002.pdf]

# Expanded View Figures

**Figure EV1. F4L5.13 specifically binds FZD4/LRP5 co-receptors.**

A F4L5.13 is specific for FZD4, as determined by biolayer interferometry (BLI). F4L5.13 or the isotype control molecule (100 nM) was tested for binding to immobilized recombinant FZD CRDs or the ectodomain of the unrelated receptor Her2. BLI sensors coated with Fc were used to determine the baseline response.

B Binding kinetics, as determined by BLI, for the binding of immobilized F4L5.13 to FZD4 CRD or LRP5. Experiments were performed in triplicate, and data are presented as the mean ± SD.

C F4L5.13 selectively binds to LRP5 over LRP6. LRP5/6 proteins coated on a maxisorp plate are recognized in a concentration-responsive manner by F4L5.13 but not by a non-targeting IgG control (4275). F4L5.13 binds to LRP5 with a low nanomolar EC50 value and is > 50-fold selective for LRP5 over LRP6. Data are presented as the mean ± SD from three independent experiments.

D Activation of βcatenin signaling by WNT3A, F4L5.13, or recombinant NDP (30 nM each) in HEK293T cells transfected with plasmids encoding FZD4 and/or LRP5. Values represent fold activation of LEF/TCF reporter gene. Data are presented as mean ± SEM, $n = 3$.

E Binding of F4L5.13 to the cell surface of HEK293T cells overexpressing FZD4 and LRP5 by flow cytometry. Data are representative of two experiments.

© 2021 The Authors

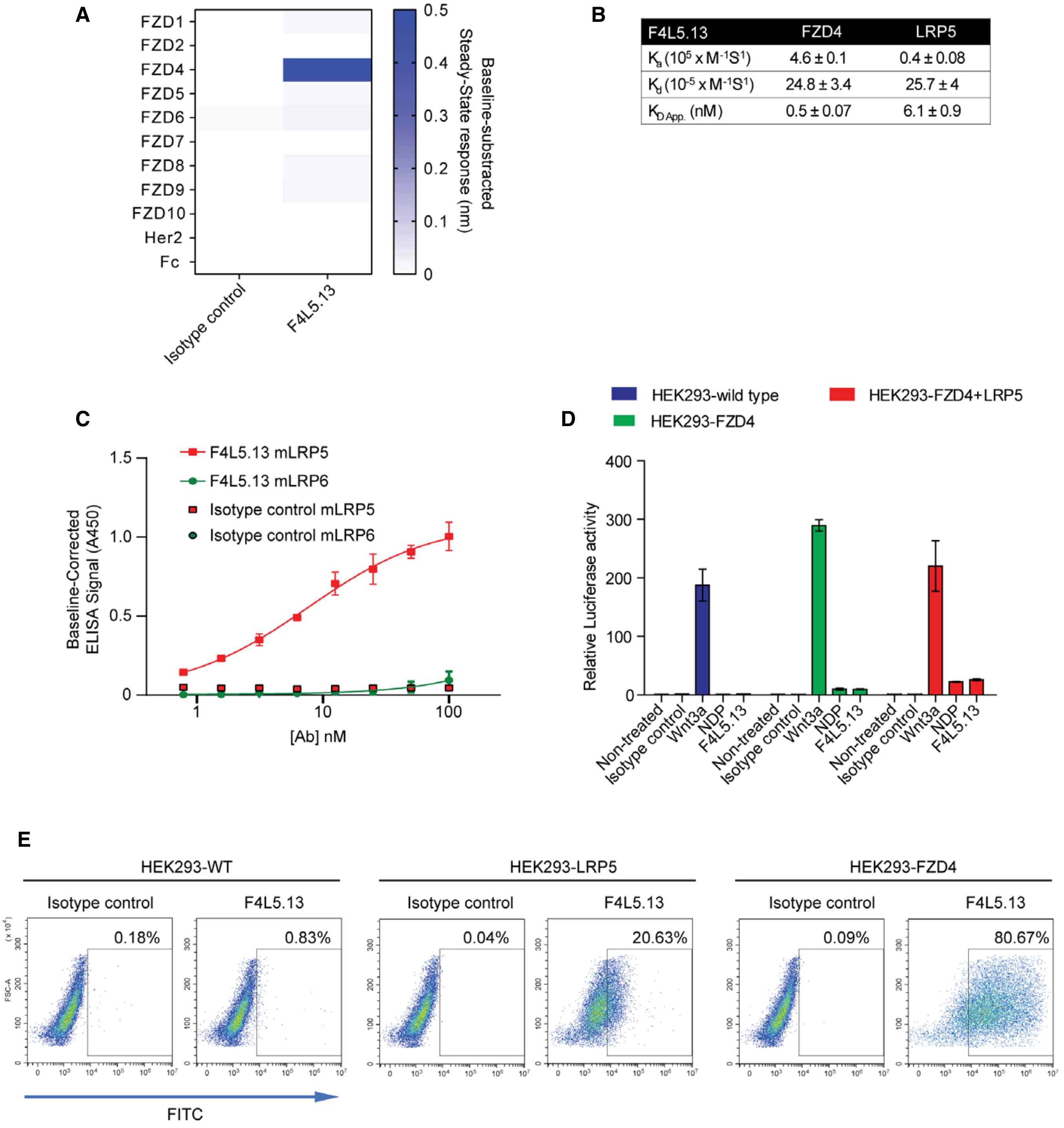


| F4L5.13 | FZD4 | LRP5 |
|---|---|---|
| $K_a$ ($10^5$ x $M^{-1}S^1$) | 4.6 ± 0.1 | 0.4 ± 0.08 |
| $K_d$ ($10^{-5}$ x $M^{-1}S^1$) | 24.8 ± 3.4 | 25.7 ± 4 |
| $K_{D App.}$ (nM) | 0.5 ± 0.07 | 6.1 ± 0.9 |

**Figure EV1.**

 © 2021 The Authors

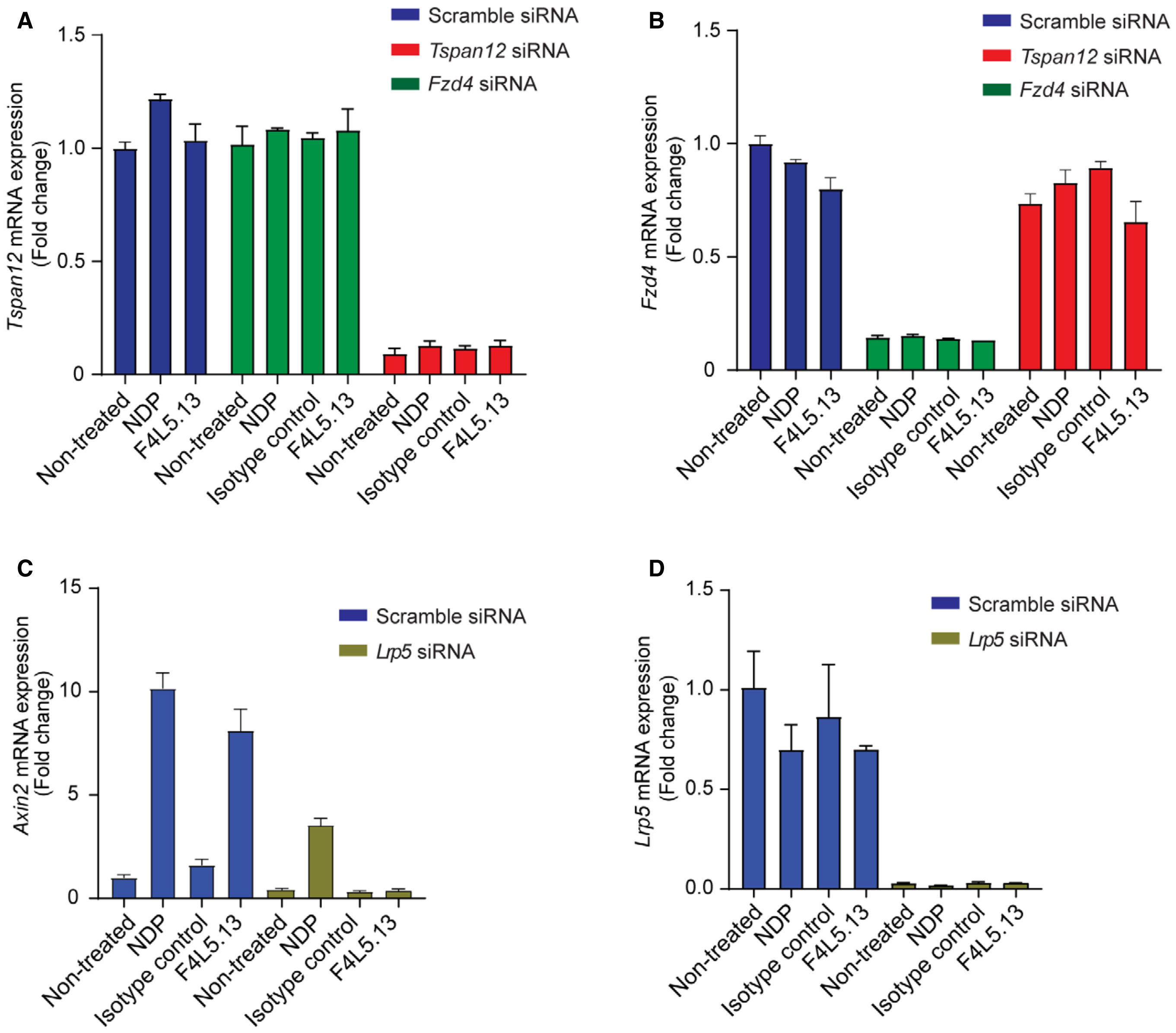


**Figure EV2. Downregulation of Tspan12, Fzd4, and Lrp5 in bEnd.3 cells.**

A, B RT–qPCR of *Tspan12* (A) and *Fzd4* (B) mRNA expression in bEnd.3 cells transfected with scrambled, *Fzd4*- or *Tspan12*-targeting siRNA and treated or not with isotype control, F4L5.13 or NDP for 24 h. Data are presented as mean ± SD, $n = 2$ technical replicates. Data are representative of two independent experiments.

C RT–qPCR of *Axin2* in bEnd.3 cells transfected with control or *Lrp5*-targeting siRNAs and treated or not with isotype control, F4L5.13 or NDP for 24 h. Data are presented as mean ± SD, $n = 3$ technical replicates. Data are representative of two independent experiments.

D RT–qPCR of *Lrp5* mRNA expression in bEnd.3 cells transfected with scrambled or *Lrp5* siRNA and treated or not with isotype control, F4L5.13 or NDP for 24 h. Data are presented as mean ± SD, $n = 3$ technical replicates. Data are representative of two independent experiments.

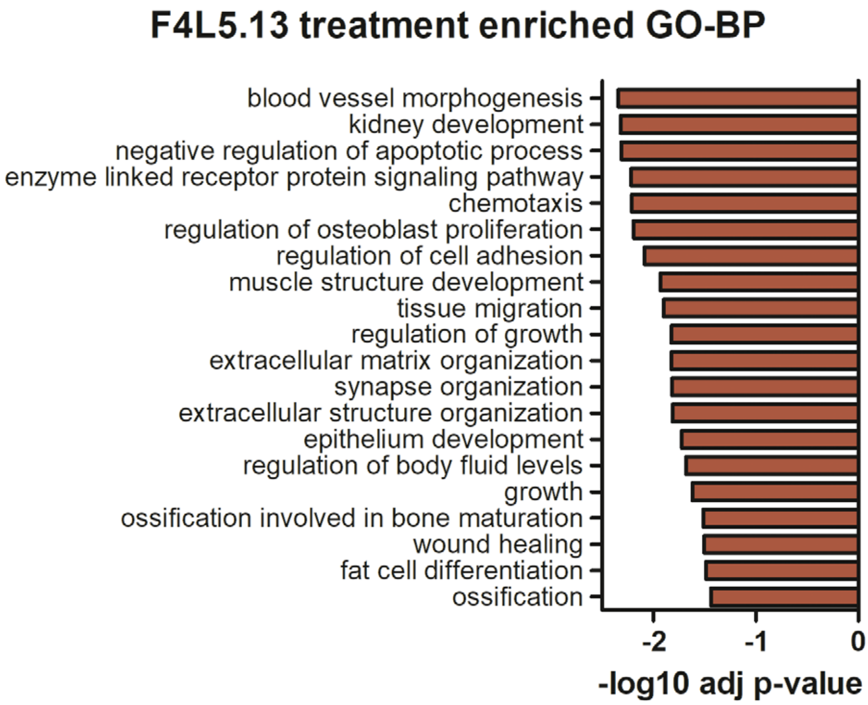


**Figure EV3. Top 20 significant GO biological processes enriched in F4L5.13-treated cells.**

Genes significantly regulated by F4L5.13 treatment were analyzed using the GO biological process annotation, and the top 20 GO terms significantly enriched (FDR adjusted *P*-value, REVIGO) were shown.

 © 2021 The Authors

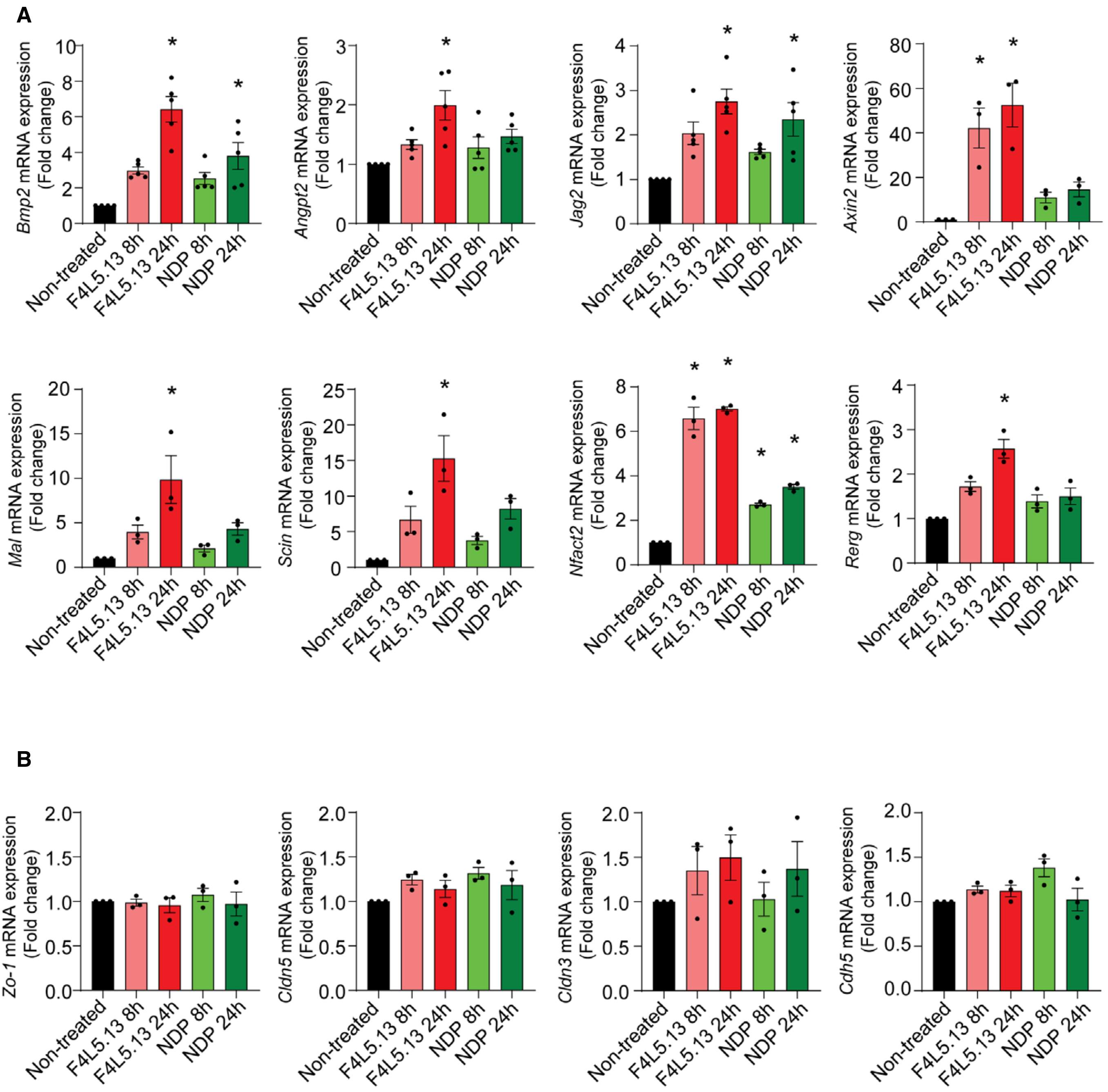


**Figure EV4. RT–qPCR validation of differentially expressed genes identified by RNA-Seq and genes involved in endothelial barrier function in bEnd.3 cells.**

A RT–qPCR of a panel of differentially expressed genes identified by RNA-Seq in bEnd.3 cells treated with F4L5.13 or NDP for 8 h and 24 h. Data are presented as mean ± SEM, $n$ = 3–5 biological replicates. Significance was calculated by one-way ANOVA with Bonferroni's multiple comparisons test (*$P$ < 0.05 as compared with NT).

B RT–qPCR of endothelial junctional genes in bEnd.3 cells treated with F4L5.13 or NDP for 8 h and 24 h. Data are presented as mean ± SEM, $n$ = 3 biological replicates. Significance was calculated by one-way ANOVA with Bonferroni's multiple comparisons test.

© 2021 The Authors
